# Bioactivity Evaluation and Phytochemical Characterization of *Euonymus alatus* (Thunb.) Siebold Leaf Extract

**DOI:** 10.3390/biomedicines12122928

**Published:** 2024-12-23

**Authors:** Won-Joo Yoon, Hye-Ji Min, Hyun-Dong Cho, Hwi-Gon Kim, Wool-Lim Park, Du-Hyun Kim, Hirofumi Tachibana, Kwon-Il Seo

**Affiliations:** 1 Division of Applied Biological Chemistry, Department of Bioscience and Biotechnology, Faculty of Agriculture, Kyushu University, Fukuoka 819-0395, Japan; wjyoon1@gmail.com (W.-J.Y.); tatibana@agr.kyushu-u.ac.jp (H.T.); 2 Department of Food Biotechnology, Dong-A University, Busan 49315, Republic of Korea; alsgpwl1473@naver.com (H.-J.M.); gnlrhs253@naver.com (H.-G.K.); dnffla9377@gmail.com (W.-L.P.); 3 Department of Food and Nutrition, Sunchon National University, Suncheon 57922, Republic of Korea; hdcho@scnu.ac.kr; 4 Department of Life Resources Industry, Dong-A University, Busan 49315, Republic of Korea; dhkimhort@dau.ac.kr

**Keywords:** *Euonymus alatus*, antioxidation, antidiabetic, anticancer, life-prolonging

## Abstract

Background: *Euonymus alatus* (*E. alatus*) has traditionally been used for medicinal purposes, and its leaves are considered edible. While *E. alatus* is known for its diverse biological activities, the antioxidant, antidiabetic, and anticancer effects of its leaves extracted using different solvents have not been thoroughly investigated. Methods: This study examined the antioxidant, antidiabetic, anticancer, and life-prolonging effects of *Euonymus alatus* (*E. alatus*) leaf extract. Results: The phytochemical analysis showed that the ethanol extract contained the highest levels of polyphenols (347.2 mg/mL) and flavonoids (317.7 mg/mL) compared to the water and methanol extracts. In addition, specific phenolics, such as rutin, ellagic acid, and quercetin, were found in the ethanol extract. Antioxidant assays showed that the ethanol extract exhibited superior DPPH, ABTS radical, and H_2_O_2_-scavenging activities, as well as reducing power. In addition, the ethanol extract displayed strong α-glucosidase inhibitory activity in a dose-dependent manner. In cancer cell studies, the ethanol extract selectively inhibited the proliferation of MDA-MB-231 (breast) and LNCaP (prostate) cancer cells without affecting normal cells. Apoptosis induction was confirmed by nuclear condensation and increased caspase-3 activity. Furthermore, treatment with 30 mg/kg/day of the extract extended the lifespan of the tumor-bearing mice to 50 days, with no fatalities, indicating a dose-dependent protective effect. Conclusions: *E. alatus* leaf ethanol extract has potential as an antioxidant, antidiabetic, anticancer, and life-prolonging agent.

## 1. Introduction

Natural products with biological activity have been used for a long time owing to their safety. In addition, they have attracted significant attention because of their diverse potential applications. In particular, plant-derived bioactive substances provide essential nutrients and contain various natural compounds, such as flavonoids, terpenoids, carotenoids, and phenolic acids [1,2]. These plant polyphenols exhibit a wide range of functional properties, including antibacterial, antioxidant, antidiabetic, and anticancer activities [3]. Furthermore, the multifunctionality and low side effects of these natural products make them attractive candidates for use in pharmaceuticals, functional foods, and cosmetics [4]. Research has revealed new mechanisms of action, expanding their potential in addressing chronic diseases and promoting health.

Aerobic metabolic processes are essential for cell survival, during which reactive oxygen species (ROS) are naturally generated [5]. These ROS play a crucial role in normal cellular functions, including cell proliferation, immune responses, and wound healing [6]. Cells have an innate antioxidant defense system to regulate these ROS levels and prevent oxidative stress. However, when the antioxidant defense system is impaired or ROS levels become excessive, oxidative stress occurs, leading to the oxidation of DNA, proteins, and membrane lipids [7,8]. This oxidative damage is closely associated with the onset and progression of several diseases, including cardiovascular diseases, neurodegenerative disorders, diabetes, and cancer.

In particular, DNA damage and genomic instability induced by oxidative stress are the critical drivers transforming normal cells into those with cancerous properties, promoting tumor initiation and progression. Moreover, these molecular alterations facilitate cancer metastasis by enhancing processes such as angiogenesis and invasiveness [9]. Considering the role of oxidative stress in disease pathologies, natural products with antioxidant properties offer promising therapeutic potential. They can mitigate oxidative stress and reduce ROS levels, making them beneficial for treating or preventing diseases such as diabetes and cancer. Bioinformatics has recently become an indispensable tool in the life sciences, providing theoretical insights into the therapeutic effects of antioxidants and targeted therapy for cancer treatment [10,11]. However, further research is required to facilitate the clinical application of these therapeutics. Ongoing research suggests that these natural antioxidants may improve the effectiveness of conventional therapies by enhancing cellular resilience and reducing treatment-related oxidative damage [12].

*Euonymus alatus* (Thunb.) Siebold (*E. alatus*) is a perennial plant belonging to the family Celastraceae that is traditionally used for m.edicinal purposes in East Asian countries [13]. Its edible leaves contain various bioactive compounds, including sesquiterpenoids, diterpenoids, triterpenoids, flavonoids, and lignans [14]. Recent studies have reported that *E. alatus* exhibits diverse biological activities, such as antitumor [15], antihypertensive [16], antidiabetic [17], and immune-modulating effects [18]. In particular, the anti-tumor effects of *E. alatus* extracts obtained using various solvents such as water, ethanol, and methanol have been reported in lung, liver, and cervical cancers at concentrations of 20–200 µg/mL [13]. Despite its potential health benefits, no comprehensive study has been conducted to compare the efficacy of its extracts using different solvents. Additionally, previous studies have not investigated the apoptotic effects of *E. alatus* leaves on breast and prostate cancer cells or its anticancer activity in an in vivo sarcoma-180 mouse ascites tumor model. Considering that the solvent polarity can influence the extraction of specific bioactive compounds, it is crucial to evaluate how different solvents affect the physiological activities of *E. alatus* leaves. Therefore, this study examined the biological properties of *E. alatus* water, ethanol, and methanol extracts including their antioxidant, antidiabetic, and anticancer effects, using both in vitro and in vivo models. This paper provides fundamental data supporting the development of *E. alatus* as a functional food ingredient, offering insight into which extraction methods yield optimal health benefits.

## 2. Materials and Methods

### 2.1. Sample Preparation

*E. alatus* leaves were collected from the Forest Bioresources Department (37°15′6″ N 126°57′32″ E), National Institute of Forest Science (NiFoS), Suwon, Republic of Korea, in November 2019, and authenticated by Dr. Hyo-In Lim, NiFoS. A voucher specimen was deposited in the Dong-A University Herbarium (No. 191107). The harvested *E. alatus* leaves were dried overnight at 65 °C and then powdered for further use. The solvents used in this study were triple distilled water, certified grade ethanol (Daejung Chemicals & Metals Co., Siheung-si, Republic of Korea), and ultrapure grade methanol (Daejung Chemicals & Metals Co., Siheung-si, Republic of Korea). The *E. alatus* water extract (EW) was prepared by mixing the plant material with water at a 1:15 (*w*/*v*) ratio and refluxing it three times for 2 h each. The ethanol extract (EE) and methanol extract (EM) were similarly prepared by mixing the material with each solvent at the same 1:15 (*w*/*v*) ratio, followed by extraction at room temperature for 24 h, repeated three times. Each extract was filtered using Whatman #2 filter paper. The water extract was freeze-dried (FD8508, IlShinBioBase Co., Dongducheon, Republic of Korea), while the ethanol and methanol extracts were concentrated using a rotary evaporator (N-1000, Eyela, Tokyo, Japan). The yield of each extract was 31% for EW, 18% for EE, and 27% for EM. The dried extracts (EE, EW, and EM) were stored at −80 °C until the experiment. Prior to use, the EW, EE, and EM were diluted with DMSO (Sigma-Aldrich Co., St. Louis, MO, USA).

### 2.2. Total Polyphenol Content Analysis

The total polyphenol content of the EW, EE, and EM was measured using a modified Folin–Ciocalteu method [19]. A reaction mixture was prepared by combining 5 μL of the sample, 45 μL of distilled water, 5 μL of 2N Folin–Ciocalteu reagent (Sigma-Aldrich Co., St. Louis, MO, USA), and 100 μL of 7% Na_2_CO_3_ (Junsei Chemical Co., Tokyo, Japan). The mixture was allowed to react for 1 h at room temperature. After the reaction, the absorbance was measured at 760 nm using a spectrophotometric microplate reader (Molecular Devices, San Jose, CA, USA). The polyphenol content was quantified based on a standard curve prepared with gallic acid (Junsei Chemical Co., Tokyo, Japan), and the results were expressed as milligrams of gallic acid equivalents per gram of extract (mg GAE/g).

### 2.3. Total Flavonoid Content Analysis

The total flavonoid content of the EW, EE, and EM was determined using a modified version of the method described by Gragaham [20]. To prepare the reaction mixture, 20 μL of sample was mixed with 200 μL of 10% diethylene glycol (Sigma-Aldrich Co., St. Louis, MO, USA) and 1 N NaOH (Daejung Chemicals & Metals Co., Siheung-si, Republic of Korea). The mixture was incubated at 37 °C for 1 h. After incubation, the absorbance was measured at 420 nm using a microplate reader (Molecular Devices, San Jose, CA, USA). The quantification was performed using a standard curve prepared with quercetin (Sigma-Aldrich Co., St. Louis, MO, USA), and the results were expressed as milligrams of quercetin equivalents per gram of extract (mg QE/g).

### 2.4. HPLC Analysis

The bioactive components of the EE were analyzed using high-performance liquid chromatography (HPLC; Shimadzu Co., Kyoto, Japan). Before analysis, the sample dissolved in DMSO was filtered through a 0.45 μm PVDF filter (Avantor Inc., Radnor, PA, USA). Separation was performed using a Phenomenex Luna C18 column (4.6 × 250 mm, 5 μm) with a mobile phase consisting of 1% acetic acid (A) and methanol (B). The flow rate was maintained at 1 mL/min, with the column temperature set to 30 °C. The injection volume was 10 μL, and the total analysis time was 40 min. The mobile phase gradient was programmed as follows: 95% solvent A and 5% solvent B from 0 to 10 min; 70% solvent A and 30% solvent B at 20 min; 10% solvent A and 90% solvent B at 30 min; 70% solvent A and 30% solvent B at 37 min; and, finally, re-equilibration to 95% solvent A and 5% solvent B at 40 min. Detection was carried out using a UV-Vis detector at wavelengths of 260 nm and 360 nm. A total of 5 injections were performed for each analysis, including 4 standard solutions (Rutin (CFN99642, ChemFaces Biochemical Co., Wuhan, China), ellagic acid (CFN98716, ChemFaces Biochemical Co., Wuhan, China), quercetin (CFN99272, ChemFaces Biochemical Co., Wuhan, China), and kaempferol (CFN98838, ChemFaces Biochemical Co., Wuhan, China)), and 1 experimental sample. Bioactive compounds were identified by comparing their retention times with those of standard compounds. Quantification was performed using calibration curves constructed from the standard solutions. The analysis method was based on previously validated protocols from Zhao et al. [21] and Pi et al. [22].

### 2.5. Assessment of Antioxidant Activity

The DPPH radical scavenging activity of EW, EE, and EM was measured using the Blois method [23]. The antioxidant efficacy was confirmed by assessing the reducing property of DPPH (α,α’-diphenyl-β-picrylhydrazin, Alfa Aesar, Haverhill, MA, USA). Specifically, 200 μL of DPPH solution was reacted with 40 μL of *E. alatus* leaf extract, and the absorbance was measured by a microplate reader (Molecular Devices, San Jose, CA, USA) at 517 nm. The results were expressed as the percentage decrease in absorbance relative to the control.

The ABTS radical scavenging activity of the EW, EE, and EM was evaluated using the method of Biglari et al. [24]. A solution of 7.4 mM 2,2′-Azobi(2-aminopropane) dihydrochloride (ABTS, Sigma-Aldrich Co., St. Louis, MO, USA) and 2.6 mM potassium peroxodisulfate (Sigma-Aldrich Co., St. Louis, MO, USA) was prepared to generate ABTS radicals in the dark for 16 h. Following this, 5 μL of *E. alatus* leaf extract was mixed with 195 μL of ABTS solution, and the absorbance was measured by a microplate reader (Molecular Devices, San Jose, CA, USA) at 734 nm after the reaction. The ABTS radical scavenging activity was quantified as the percentage decrease in absorbance compared to the control.

The reducing power of the EW, EE, and EM was determined by modifying the method of Yildirim et al. [25]. A mixture of 50 μL of each sample, 250 μL of phosphate buffer (0.2 M, pH 6.6), and 250 μL of 1% potassium ferricyanide (Junsei Chemical Co., Tokyo, Japan) was incubated at 50 °C for 30 min. The reaction was halted by adding 250 μL of 10% trichloroacetic acid (TCA, Sigma-Aldrich Co., St. Louis, MO, USA), followed by centrifugation at 3000 rpm for 10 min. To the supernatant, 250 μL of distilled water and 50 μL of 0.1% FeCl_3_ (Sigma-Aldrich Co., St. Louis, MO, USA) were added, and the absorbance was measured by a microplate reader (Molecular Devices, San Jose, CA, USA) at 700 nm.

The hydrogen-peroxide-scavenging activity of the EW, EE, and EM was assessed using the method of Műler [26]. In this assay, 20 μL of hydrogen peroxide (Sigma-Aldrich Co., St. Louis, MO, USA) was added to 100 μL of the sample. After incubating at 37 °C for 5 min, 30 μL each of 1.25 mM ABTS and peroxidase (Sigma-Aldrich Co., St. Louis, MO, USA) were mixed into the reaction solution, which was further incubated at 37 °C for 10 min. The absorbance was measured by a microplate reader (Molecular Devices, San Jose, CA, USA) at 405 nm. The hydrogen-peroxide-scavenging activity of the *E. alatus* leaf extract was evaluated by comparing the absorbance to the control.

All antioxidant experiments used 0.1% α-tocopherol (Sigma-Aldrich Co., St. Louis, MO, USA) and 0.1% BHT (Sigma-Aldrich Co., St. Louis, MO, USA) as positive controls.

### 2.6. α-Glucosidase Inhibitory Activity

The α-glucosidase inhibitory activity of the EW, EE, and EM was measured using the method of Kim et al. [27]. The sample was mixed with 50 μL of 0.2 unit/mL α-glucosidase (Sigma-Aldrich Co., St. Louis, MO, USA) and 200 mM potassium phosphate buffer (Daejung Chemicals & Metals Co., Siheung-si, Republic of Korea), and the mixture was incubated at 37 °C for 10 min. Following this, 100 μL of 3 mM pNPG (p-nitrophenyl-α-D-glucopyranoside, Sigma-Aldrich Co., St. Louis, MO, USA) was added and the reaction continued at 37 °C for an additional 10 min. The reaction was stopped by adding 750 μL of 0.1 M sodium carbonate (Junsei Chemical Co., Tokyo, Japan), and the absorbance was measured using a microplate reader (Molecular Devices, San Jose, CA, USA) at 405 nm. The α-glucosidase inhibitory activity was expressed as the percentage decrease in absorbance compared to the control, and compared with acarbose (Sigma-Aldrich Co., St. Louis, MO, USA), an α-glucosidase inhibitor.

### 2.7. Cell Culture

A lung cancer cell line (A549), liver cancer cell line (HepG-2), prostate cancer cell lines (LNCaP-FGC (LNCaP) and PC3), prostate normal cell line (RWPE-1), breast cancer cell lines (MCF-7 and MDA-MB-231), breast normal cell line (HMEC), and murine sarcoma cell line (Sarcoma-180) were used, all of which were purchased from the American Type Culture Collection (ATCC, Manassas, VA, USA). All cells were cultured in Keratinocyte SFM (RWPE-1, Gibco, Thermo Scientific Co., Waltham, MA, USA), Mammary Epithelial Cell Growth Medium (HMEC, Lonza Inc., Basel, Switzerland), DMEM (HepG-2, LNCaP, and PC3, Gibco, Thermo Scientific Co., Waltham, MA, USA), and RPMI 1640 (A549, MCF-7, MDA-MB-231, and Sarcoma-180, Gibco, Thermo Scientific Co., Waltham, MA, USA) containing 10% FBS (Gibco, Thermo Scientific Co., Waltham, MA, USA) and 1% penicillin/streptomycin (Gibco, Thermo Scientific Co., Waltham, MA, USA). The cells were maintained in a CO_2_ incubator (SCA-165DS, ASTEC, Fukuoka, Japan) at 37 °C with 5% CO_2_.

### 2.8. SRB (Sulforhodamine B) Assay

The cell proliferation inhibitory activity was evaluated using the SRB assay. A549, LNCaP, HepG-2, MDA-MB-231, PC3, MCF-7, RWPE-1, and HMEC cells were seeded into 48-well plates at a density of 3 × 10^4^ cells/mL and stabilized for 24 h. The samples (EW, EE, and EM) were applied at concentrations ranging from 1 µg/mL to 30 µg/mL, with a control group included. After 24 h of incubation, the medium was removed, and the cells were fixed with 10% TCA. The fixed cells were then stained with 0.4% SRB (Sigma-Aldrich Co., St. Louis, MO, USA). Following the staining process, the cells were washed with 1% acetic acid (Daejung Chemicals & Metals Co., Siheung-si, Republic of Korea), and 10 mM Tris (BioShop Canada Inc., Burlington, ON, Canada) buffer was added to dissolve the bound SRB dye. The absorbance was measured at 540 nm using a microplate reader (Molecular Devices, San Jose, CA, USA) to assess the cell proliferation inhibitory activity.

### 2.9. Morphological Analysis

For morphological observations, LNCaP, MDA-MB-231, PC3, MCF-7, RWPE-1, and HMEC cells were seeded at a density of 3 × 10^4^ cells/mL in 6-well plates and treated with 30 µg/mL of EE for 24 h. After treatment, morphological changes were observed using an optical microscope (Leica Microsystems, Wetzlar, Germany), and images were captured.

### 2.10. Hoechst Staining

Breast cancer cells (MDA-MB-231) and prostate cancer cells (LNCaP) were seeded at a density of 3 × 10^4^ cells/mL in 6-well plates and then treated with 15 and 30 µg/mL of EE for 24 h. After treatment, 2 μg/mL Hoechst 33258 (bis-benzimide, Sigma-Aldrich Co., St. Louis, MO, USA) was added to the harvested cells and stained for 20 min. The stained cells were observed using a confocal microscope (Olympus Optical Co., Ltd., Tokyo, Japan).

### 2.11. Caspase-3 Activity

Caspase-3 activity was measured using the Caspase-3 Activity Assay Kit (Cell Signaling Technology, Danvers, MA, USA) following the manufacturer’s instructions. After treating MDA-MB-231 and LNCaP cells with the *E. alatus* ethanol extract at concentrations of 15 and 30 μg/mL, the cells were harvested. A lysis buffer was added to the cell pellet, followed by sonication on ice. The mixture was centrifuged at 1000× *g* for 10 min, and the supernatant was collected as the cell lysate. Ac-DEVD-AMC was added to the lysate, and after 1 h of incubation, fluorescence intensity was measured with a spectrophotometer (VANTAstar, BMGLABTECH, Ortenberg, Germany) using an excitation wavelength of 380 nm and an emission wavelength of 460 nm, to assess the caspase-3 activity.

### 2.12. Western Blot

MDA-MB-231 and LNCaP cells were seeded at a density of 3 × 10^4^ cells/well. After 24 h, the cells were treated with EE at concentrations of 10, 15, and 30 μg/mL for 24 h. Following treatment, proteins were extracted using a cell lysis buffer and quantified using a BCA protein assay kit (Thermo Scientific Co., Waltham, MA, USA). The proteins were separated by SDS-PAGE and transferred onto polyvinylidene fluoride (PVDF) membranes (Cytiva, Little Chalfont, UK). The membranes were then probed with primary and secondary antibodies, and protein detection was performed using an ECL kit (Cytiva, Little Chalfont, UK). Protein band intensities were quantified using ImageJ software (v. 1.52a, NIH, Bethesda, MD, USA), and changes in protein expression levels were normalized to β-actin. Anti-caspase-3 (sc7272) and anti-β-actin (sc-47778) were obtained from SantaCruz Biotechnology (Santa Cruz, CA, USA).

### 2.13. Extension of Lifespan of Tumor-Bearing Mice

Four-week-old male ICR mice used in the experiment were housed in acrylic cages at 22 ± 2 °C with a 12-h light/dark cycle. The mice were randomly divided into three groups of eight. After a one-week acclimation period, each mouse was inoculated intraperitoneally with 1 × 10⁶ Sarcoma-180 cells. At 24 h post-inoculation, samples were administered intraperitoneally for seven consecutive days. The control group received sterilized distilled water, while the experimental groups received 100 μL of EE (dissolved in sterilized distilled water) at doses of 15 mg/kg/day and 30 mg/kg/day, respectively. The survival of the mice was monitored for up to 50 days. All animal experiments were performed in accordance with the Dong-A University Laboratory Animal Care and Use Guidelines (DIACUC-24-49).

### 2.14. Statistical Analysis

All experiments were performed at least three times. Data are expressed as means ± SD. Statistical significance was determined using one-way ANOVA followed by Duncan’s multiple test or Dunnett’s test at * *p* < 0.05, ** *p* < 0.01, *** *p* < 0.001. Statistical analysis was conducted using the Statistic Analysis System (8.01, SAS Institute Inc., Cary, NC, USA) and GraphPad Prism (v. 5.0, GraphPad Software, San Diego, CA, USA).

## 3. Results

### 3.1. Pytochemical Profiles of E. alatus Extracts and Bioactive Compound Identification in EE

The total polyphenol and flavonoid contents were measured to confirm the antioxidant activity of the *E. alatus* leaf extract (Table 1). Polyphenols and flavonoids were quantified using gallic acid and quercetin as standards, respectively. Among the extracts, EE showed the highest polyphenol content at 347.2 mg GAE/g, followed by EM at 328.4 mg GAE/g and EW at 312.9 mg GAE/g. Similarly, the total flavonoid content followed the same trend, with EE yielding the highest amount. Statistical analysis showed significant differences between the extracts, confirming that ethanol is the most effective solvent for extracting flavonoids and polyphenols from *E. alatus* leaves.

**Figure 1:**
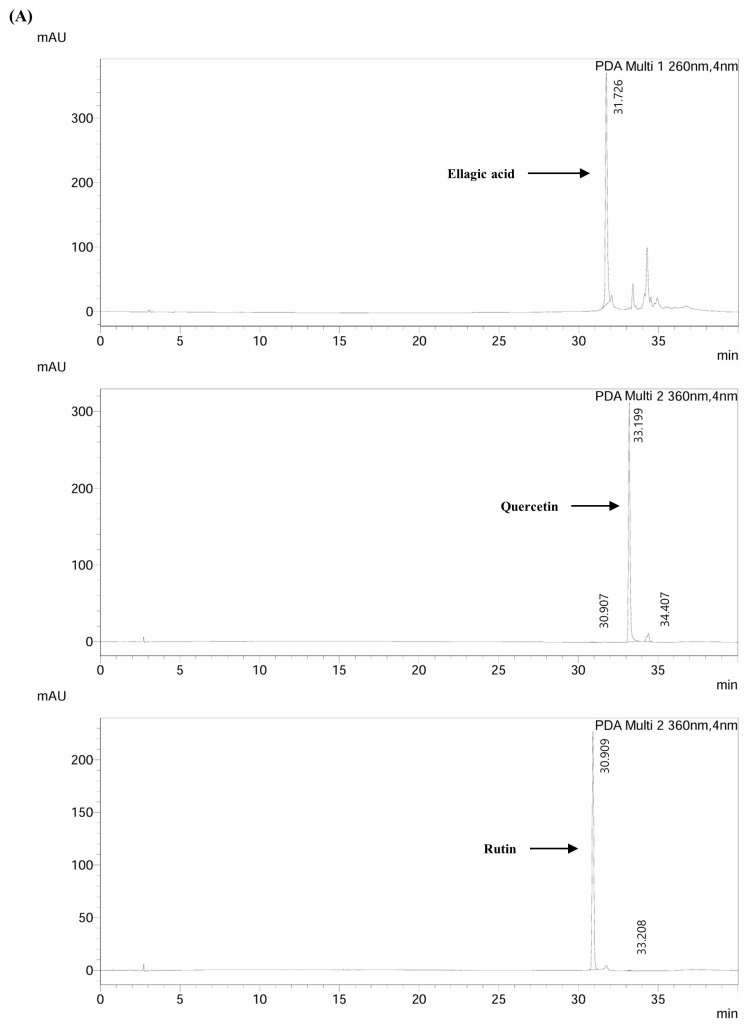

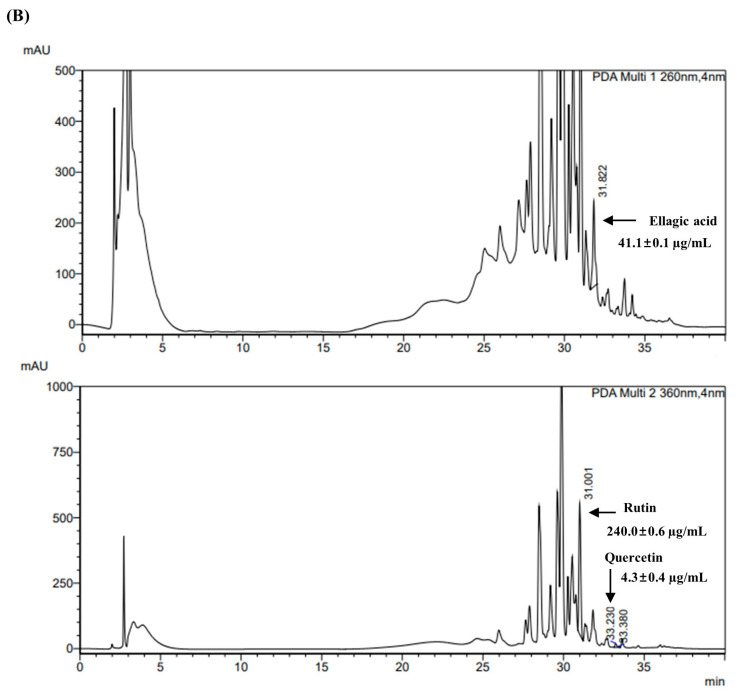
Chromatography of phenolic compounds in *E. alatus* leaf ethanol extract (EE). (**A**) Representative chromatograms of phenolic compound standards at 260 and 360 nm. (**B**) High-performance liquid chromatography (HPLC) was used for the analysis of phenolic compounds in EE at 260 and 360 nm of wavelength. The results are shown as the mean ± standard deviation of triplicate determinations.

Subsequently, high-performance liquid chromatography (HPLC) was used to identify the bioactive compounds in the EE (Figure 1). Ellagic acid (41.11 mg/mL), rutin (240.02 mg/mL), and quercetin (4.258 mg/mL) were present, with rutin being the most abundant. The high rutin content may contribute significantly to the potent antioxidant properties of EE.

### 3.2. Antioxidant Activity of E. alatus Leaf Extract

**Figure 2:**
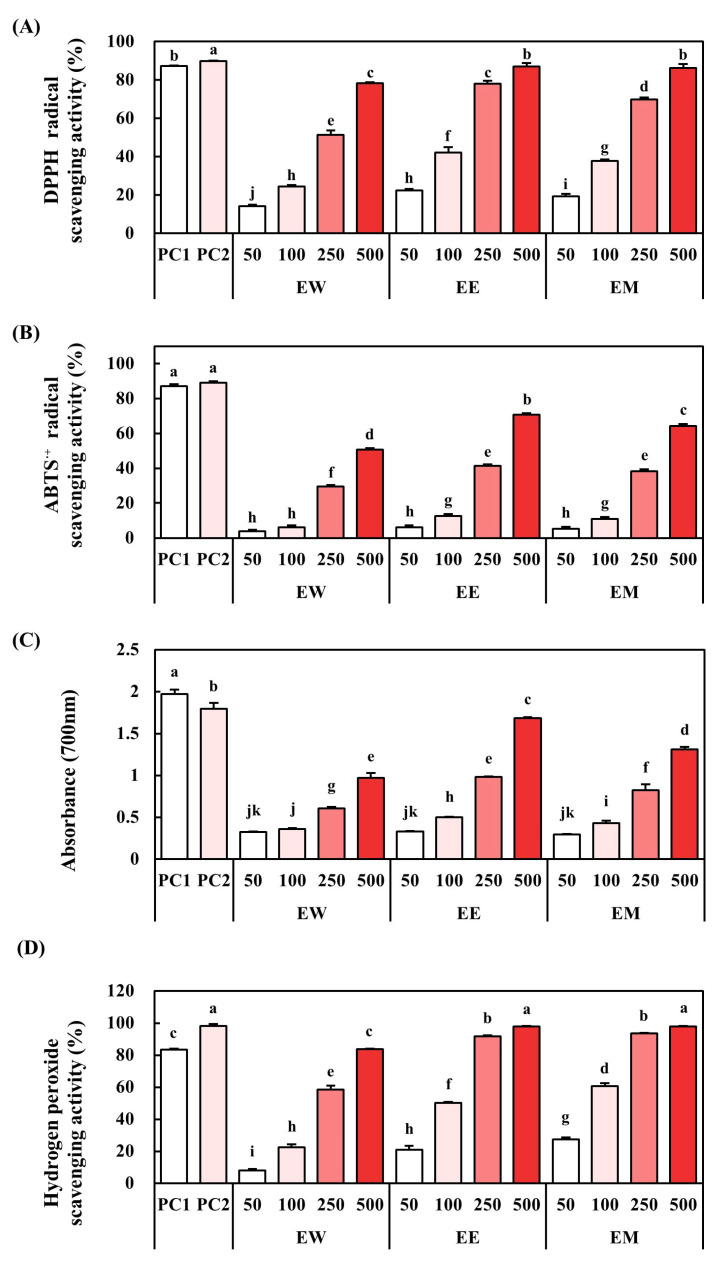
Changes in the antioxidant effects of *E. alatus* extracts by solvents. (**A**) DPPH-radical-scavenging activity (%), (**B**) ABTS^·+^-radical-scavenging activity (%), (**C**) reducing power, (**D**) hydroxyl-radical-scavenging activity (%). PC1, 0.1% α-tocopherol; PC2, 0.1% BHT; EW, *E. alatus* leaf water extracts (μg/mL); EE, *E. alatus* leaf ethanol extract (μg/mL); EM, *E. alatus* leaf methanol extracts (μg/mL). The results are presented as the mean ± SD of triplicate determinations. Different lowercase letters were determined using Duncan’s multiple range test (*p* < 0.05) and indicate differences between each group.

The antioxidant activity of the *E. alatus* leaf extract was evaluated through its DPPH radical scavenging activity, ABTS-radical-scavenging activity, reducing power, and hydrogen-peroxide-scavenging activity. The results are presented in Figure 2.

The DPPH assay measures the ability of a sample to donate hydrogen atoms and neutralize the DPPH radical, a relatively stable free radical. Among the extracts, EE exhibited the highest DPPH radical scavenging activity, comparable to the positive controls, to 0.1% BHT and α-tocopherol. EM also exhibited significantly lower DPPH-scavenging ability than EE, while EW showed the least activity.

Similarly, the ABTS radical scavenging assay, which measures the ability to suppress ABTS radicals, showed that EE exhibits the highest activity. These results align with the DPPH scavenging results, underscoring the efficacy of EE in scavenging free radicals.

The reducing power assay evaluates the electron-donating capacity of the extracts, which is one of the critical antioxidant mechanisms. The *E. alatus* extracts showed concentration-dependent increases in reducing power. Although EE showed slightly lower activity than the positive control, it exhibited the highest absorbance among the tested extracts.

Hydrogen peroxide (H_2_O_2_) plays an essential role in cell signaling pathways, but is also classified as an ROS. Excessive H_2_O_2_ can cause oxidative stress, leading to cellular damage [28]. In the H_2_O_2_ scavenging assay, EM showed the highest activity, but was similar to EE. These findings showed that *E. alatus* leaf extracts possess strong antioxidant activity, with EE consistently showing the highest performance across multiple assays.

### 3.3. α-Glucosidase Inhibitory Activity of E. alatus Leaf Extract

**Figure 3:**
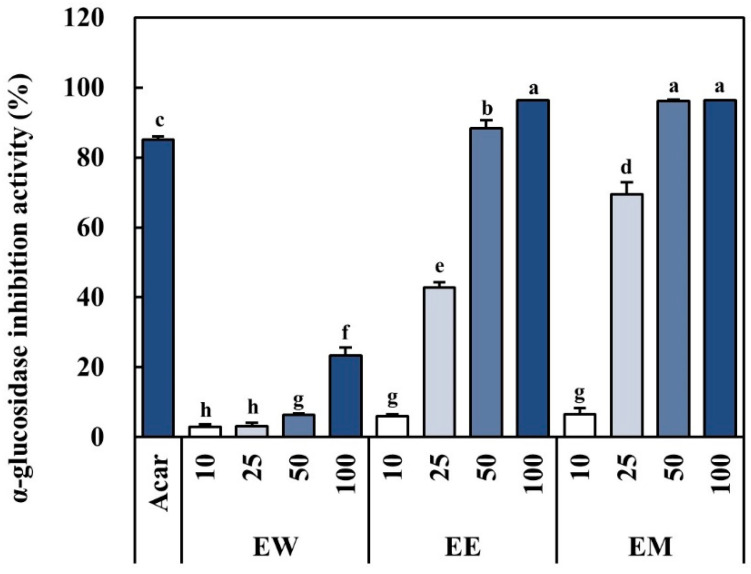
Changes in α-glucosidase activities of *E. alatus* extracts by different solvents. Acar, 1% acarbose; EW, *E. alatus* leaf water extracts (μg/mL); EE, *E. alatus* leaf ethanol extract (μg/mL); EM, *E. alatus* leaf methanol extracts (μg/mL). The results are expressed as the mean ± SD from three independent experiments. Different lowercase letters were determined using Duncan’s multiple range test (*p* < 0.05) and indicate differences between each group.

An α-glucosidase inhibition assay was performed to investigate the effect of *E. alatus* leaf extract on α-glucosidase inhibition activity (Figure 3). The inhibition activity increased in a concentration-dependent manner across all extracts. In particular, EE and EM showed comparable or superior efficacy to the positive control, 1% acarbose, whereas EW exhibited relatively low inhibitory activity. The regulation of α-glucosidase can influence diabetes by controlling postprandial blood glucose levels. These findings suggest that EE has potential for glycemic control.

### 3.4. Inhibition of Cancer Cell Proliferation by E. alatus Leaf Extract

**Figure 4:**
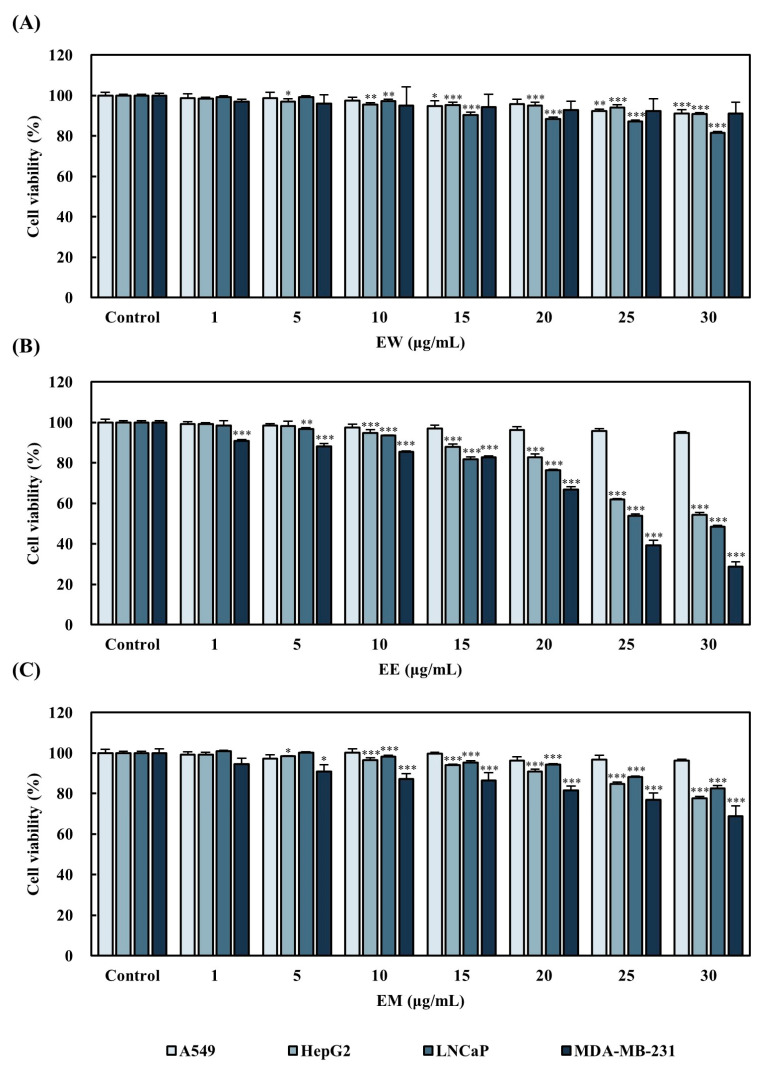
Effects of *E. alatus* extracts by different solvents on A549, HepG2, LNCaP, and MDA-MB-231 cell lines after 24 h of treatment. (**A**) Treatment of *E. alatus* leaf water extract on A549, HepG2, LNCaP, and MDA-MB-231 cell lines for 24 h; (**B**) treatment of *E. alatus* leaf ethanol extract on A549, HepG2, LNCaP, and MDA-MB-231 cell lines for 24 h; (**C**) treatment of *E. alatus* leaf methanol extract on A549, HepG2, LNCaP, and MDA-MB-231 cell lines for 24 h. The results are expressed as a percentage relative to the control for each cell line and presented as the mean ± SD from three independent experiments. Statistical significance was determined using one-way ANOVA followed by Dunnett’s test. * *p* < 0.05, ** *p* < 0.01, *** *p* < 0.001 indicate significant differences from the control group.

The cancer cell proliferation inhibitory effect of *E. alatus* leaf extract was examined by treating various cancer cell lines with the extracts for 24 h. EW did not significantly reduce cell viability (Figure 4). On the other hand, EE and EM effectively inhibited cancer cell survival. In particular, EE exhibited the largest decrease in cell viability, particularly in HepG2 (liver cancer), LNCaP (prostate cancer), and MDA-MB-231 (breast cancer) cells. These findings show that EE is the most potent in inhibiting cancer cell proliferation; hence, it was selected for subsequent experiments.

### 3.5. Cancer Cell Specific Inhibition of EE

Treatment with EE resulted in a specific decrease in the viability of certain cancer cell lines, particularly breast and prostate cancer cells. This effect was confirmed by further experiments using different breast cancer cells, prostate cancer cells, and corresponding normal cell lines.

**Figure 5:**
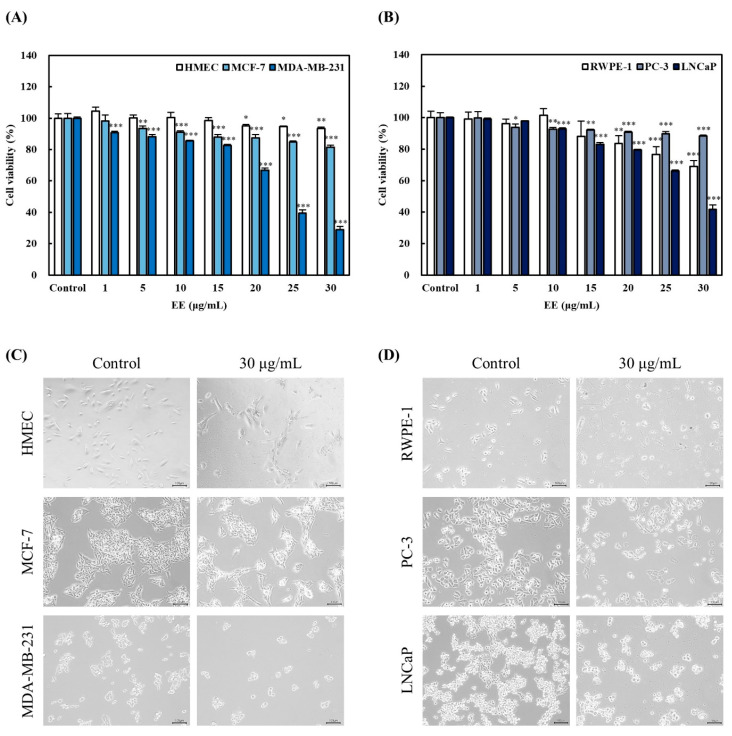
Effects of EE on cell viability in breast and prostate tissue cells. Effects of EE on the cell viability of (**A**) HMEC, MCF-7, and MDA-MB-231 cells and (**B**) RWPE-1, PC-3, and LNCaP cells for 24 h. Morphological changes were observed for 24 h in (**C**) HMEC, MCF-7, and MDA-MB-231 and (**D**) RWPE-1, PC-3, and LNCaP at the concentration of 30 µg/mL. EE, *E. alatus* leaf ethanol extract (μg/mL). The results are expressed as a percentage relative to the control for each cell line and presented as the mean ± SD from three independent experiments. Statistical significance was determined using one-way ANOVA followed by Dunnett’s test. * *p* < 0.05, ** *p* < 0.01, *** *p* < 0.001 indicate significant differences from the control group. Morphological changes were captured by confocal microscopy (×10) and the length of the scale bar is equivalent to 100 μm.

EE significantly inhibited the viability of MDA-MB-231 (breast cancer) and LNCaP (prostate cancer) cells in a dose-dependent manner (Figure 5A,B). On the other hand, no substantial inhibitory effect was observed in MCF-7 (breast cancer) or PC-3 (prostate cancer) cells. Furthermore, the extract did not affect the viability or morphological characteristics of HMEC (normal breast epithelial cells). To further confirm the occurrence of morphological changes, we examined cancer and normal cells at the most effective concentration (30 μg/mL). Although some mild toxicity was observed in RWPE-1 (normal prostate epithelial cells), it was significantly lower than in LNCaP cancer cells (Figure 5C,D). These findings suggest that EE selectively inhibits the proliferation of MDA-MB-231 and LNCaP cells, while showing minimal toxicity toward corresponding normal cells. This selective action highlights its potential as a therapeutic agent targeting specific cancer cell types. Subsequent experiments were conducted using concentrations of 15 and 30 µg/mL, which were selected to evaluate their overall inhibitory effects on cell viability.

### 3.6. Effect of EE on the Induction of Apoptosis

**Figure 6:**
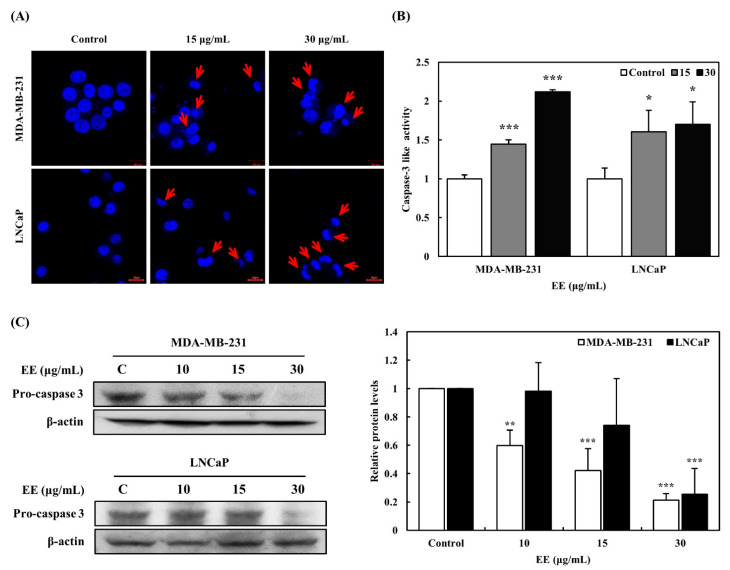
Effects of EE on apoptosis of breast and prostate cancer cells. (**A**) Nuclear staining of MDA-MB-231 and LNCaP treated with EE (×40). Cells were stained with Hoechst 33342. The scale bar indicates 25 μm. The arrows indicate areas suspected to be apoptotic body formation. (**B**) Effects of EE on caspase-3 like activity in MDA-MB-231 and LNCaP cells. (**C**) Expression of pro-caspase 3 proteins was detected by Western blotting analysis. EE, *E. alatus* leaf ethanol extract (μg/mL). The results are presented as mean ± SD based on three independent experiments. Each experiment’s results were reported relative to the control group. Statistical significance was determined using one-way ANOVA followed by Dunnett’s test. * *p* < 0.05, ** *p* < 0.01, *** *p* < 0.001 indicate significant differences from the control group.

Hoechst staining, caspase-3 activity assays, and Western blot analysis were conducted to determine whether the EE-induced inhibition of cancer cell growth was associated with apoptosis (Figure 6). Cancer cells have a characteristic tendency to suppress apoptosis, and controlling this process could be an effective cancer treatment strategy [29]. Apoptotic cell death is typically characterized by nuclear condensation, caspase activation, and cell membrane blebbing. After treating MDA-MB-231 (breast cancer) and LNCaP (prostate cancer) cells with EE, nuclear condensation and membrane blebbing were observed dose-dependently (Figure 6A). In addition, caspase-3 activity, an essential indicator of apoptosis, was measured using a caspase-3 activation kit. Treatment with EE dose-dependently activated caspase-3 in MDA-MB-231 breast cancer cells at concentrations of 15 and 30 μg/mL (Figure 6B). Although LNCaP cells did not show the dose-dependent activation of caspase-3, its levels were also significantly upregulated compared to the control group. Furthermore, the Western blot analysis showed a dose-dependent decrease in pro-caspase 3 levels in MDA-MB-231 cells, with significant reductions observed at 15 and 30 μg/mL after EE treatment (Figure 6C). In LNCaP cells, pro-caspase 3 levels were reduced, but statistically significant changes were only observed at 30 μg/mL. These results indicate that caspase-3 activation is more pronounced in MDA-MB-231 cells compared to LNCaP cells. Taken together, these findings suggest that EE induces apoptosis in MDA-MB-231 and LNCaP cells, primarily through a caspase-dependent pathway, although the effect is more pronounced in MDA-MB-231 cells.

### 3.7. Effect of EE on Life Extension in Tumor-Bearing Mouse Model

**Figure 7:**
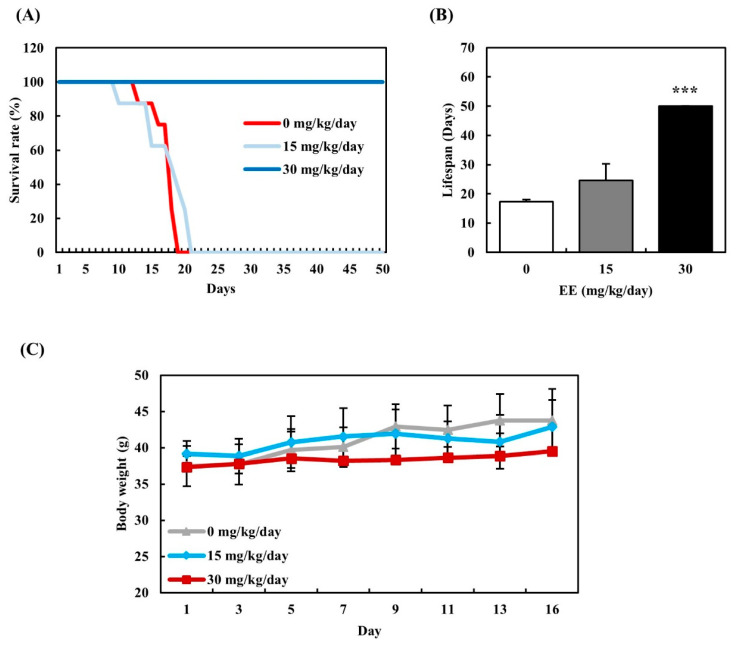
Effects of EE on lifespan extension in tumor-bearing mouse model. (**A**) Survival rates expressed by Kaplan–Meier analysis and (**B**) average lifespan were compared in all groups. (**C**) Changes in body weight were measured in all groups for 16 days. Mice were inoculated intraperitoneally with Sarcoma-180 ascite tumor cells and EE were administered for seven consecutive days at the concentrations of 15 mg and 30 mg/kg/day. EE, *E. alatus* leaf ethanol extract. Statistical significance was determined using one-way ANOVA followed by Dunnett’s test. *** *p* < 0.001 indicate significant differences from the control group.

The anticancer activity of EE against Sarcoma-180 ascites tumors was evaluated in vivo (Figure 7). In the control group, the mean survival time was 17.4 days before all subjects succumbed. By contrast, when treated with EE at 15 mg/kg/day, the treated group demonstrated an average survival time of 24.6 days, representing a 41.7% increase in lifespan. Furthermore, the group treated with EE at 30 mg/kg/day had an average lifespan of 50 days, with no observed fatalities, indicating a dose-dependent life-prolonging effect in mice. We also observed the body weight change of mice during the 16-day period (Figure 2). No significant weight changes were observed in either the control or EE-treated groups. After the mice succumbed, body weight measurements were no longer taken. These results suggest that the EE extract had a protective effect against tumor-induced mortality in vivo.

## 4. Discussion

Natural products have been used for medicinal purposes throughout human history. In recent years, the development of new drugs derived from natural products has attracted considerable interest in modern medicine. Natural products containing diverse bioactive compounds play a crucial role in preventing and treating various diseases, including cancer, cardiovascular conditions, diabetes, and metabolic disorders [30]. Furthermore, the long history of safe usage suggests that these products are associated with low toxicity and fewer side effects [18].

The major bioactive compounds identified in EE (rutin, ellagic acid, and quercetin) have antioxidant, anti-inflammatory, and anticancer properties [31,32,33]. A previous study identified quercetin, kaempferol, rutin, and abruslactone in the ethanol extract of E. alatus, partially aligning with our findings [34]. Park et al. [35] identified 3,4-dihydroxycinnamic acid, a novel matrix metalloproteinase-9 inhibitor, in the methanol extract of *E. alatus*. In particular, rutin, the most abundant compound, is a quercetin glycoside that suppresses reactive oxygen species (ROS), protecting the cells from oxidative damage [36]. Rutin induces apoptosis by activating caspase-3 in several cancer cell lines [37]. These findings provide valuable data on the chemical composition and biological activities of *E. alatus* leaves, with potential applications in the development of natural pharmaceuticals. However, comprehensive research is necessary to elucidate the roles of major bioactive compounds in the anticancer effects of EE, particularly in comparison with methanol and water extracts.

The cellular antioxidant system consists of enzymatic and non-enzymatic mechanisms that help mitigate oxidative stress by neutralizing ROS [38]. Enzymatic antioxidants, such as glutathione peroxidase, catalase, and superoxide dismutase, transform ROS into less harmful substances such as water and oxygen. Non-enzymatic antioxidants, including ascorbic acid, tocopherol, polyphenols, and flavonoids, neutralize free radicals directly and are often ingested through plant-based foods [39]. This study assessed the antioxidant activity of *E. alatus* leaf extracts using DPPH, ABTS, reducing power, and H_2_O_2_ scavenging assays. EE and EM exhibited higher antioxidant activity than EW, with EE showing the highest efficacy. Since *E. alatus* extracts exhibit significant tumor inhibitory effects and contain various phenolic compounds, they indicate a broad spectrum of anticancer and antioxidant activities [13]. In the current study, the antioxidant effects of EE were consistent with Gurung et al. [34], who reported that the ethanol fraction of *E. alatus* leaves inhibited intracellular ROS production. Kwon et al. [40] reported that the hot water extract of *E. alatus* leaves exhibited antioxidant activity, despite the lower polyphenol content.

The enzyme α-glucosidase, which breaks down carbohydrates into glucose, is key in regulating postprandial blood sugar levels. Inhibiting this enzyme can slow glucose absorption, alleviate hyperglycemia, and aid in managing diabetes [41]. α-glucosidase inhibitors also have broader therapeutic potential, influencing metabolism, obesity, tumor metastasis, and viral infections [42]. Natural compounds, such as polyphenols, flavonoids, and terpenoids, have α-glucosidase inhibitory activity, offering long-term benefits with minimal side effects [43]. In this study, the EE and EM extracts of *E. alatus* leaves showed α-glucosidase inhibitory activity comparable to the positive control, 1% acarbose, while the EW showed weaker activity. Lee et al. [44] reported that 500 µg/mL of *E. alatus* methanol extract showed about 25% of α-glucosidase suppression, and Kim et al. [45] also found that quercetin derivate significantly inhibited α-glucosidase activity. Furthermore, the *E. alatus* extract alleviated the symptoms in pancreatic β-cells and type 2 diabetic mouse models [46], indicating its potential as a diabetes treatment.

Traditionally, *E. alatus* has been used to treat various diseases and has recently attracted attention for its pharmacological properties [13]. The phenolic compounds in *E. alatus* leaves reduce oxidative stress, protect normal cells from damage, and inhibit cancer cell growth [47]. EE exhibited the highest anticancer activity against breast and prostate cancer cells compared to EW and EM. Subsequent experiments focused on EE. An EE treatment inhibited the proliferation of MDA-MB-231 breast cancer cells and LNCaP prostate cancer cells without affecting normal breast cells. Although the extract exhibited some toxicity toward normal prostate epithelial cells (RWPE-1), it was less toxic to these cells than cancer cells. Inducing apoptosis is a key objective in cancer therapy because it helps eliminate mutated cells and maintain genetic stability [48]. Caspase-3, a crucial executor of apoptosis, plays a key role in cell death [49]. EE induced typical apoptotic changes, including nuclear condensation, blebbing, and cell shrinkage, as well as caspase-3 activation in MDA-MB-231 and LNCaP cells. The Sarcoma-180 (S-180) tumor-bearing model is widely used to evaluate anticancer effects because it is a transplantable tumor line with consistent growth in mice, providing a controlled and reproducible system [50]. A recent study confirmed that the fate of cancer cells can be controlled by anticancer phenolic compounds in vivo [51]. In the control group, which did not receive the extract, all mice died within 17.4 days. In contrast, mice treated with EE showed improved survival in a dose-dependent manner, with the low- and high-dose group surviving 24.6 days and up to 50 days on average, respectively. These results show that *E. alatus* leaf extract containing abundant phenolics induces apoptotic cell death in breast and prostate cancer cells while extending the lifespan in cancer-bearing mice.

In this study, we suggest that *E. alatus* leaf extract, especially the ethanol extract, possesses effective antioxidant, antidiabetic, and anticancer properties. In particular, the observed efficacy in apoptosis induction and in vivo life extension experiments in MDA-MB-231 and LNCaP cells suggests that *E. alatus* leaf has significant potential as a natural product pharmaceutical.

## 5. Conclusions

This study analyzed the biological activities of *E. alatus* leaf extracts prepared from different solvents. The extracts demonstrated significant antioxidant, antidiabetic, and anticancer properties. Notably, the ethanol extract induced apoptosis in both breast cancer (MDA-MB-231) and prostate cancer (LNCaP) cells and showed effective life extension in the sarcoma-180 mouse model. Although further research is necessary to explore the underlying mechanisms and potential clinical applications, these results suggest the promising potential of *E. alatus* leaf extract as a natural product-based functional ingredient for pharmaceuticals and health-promoting supplements.

## Figures and Tables

**Table 1:** Changes in total polyphenol and total polyphenol concentration of *E. alatus* extracts by solvent.

Content	EW	EE	EM
Total polyphenols (mg GAE/g)	312.9 ± 10.53 ^b^	347.2 ± 15.22 ^a^	328.4 ± 16.49 ^ab^
Total flavonoids (mg QE/g)	210.6 ± 2.77 ^c^	317.73 ± 5.09 ^a^	264.66 ± 13.18 ^b^

The results are shown as the mean ± standard deviation of triplicate determinations. Total polyphenols and total flavonoids were quantified using gallic acid and quercetin. EW: *E. alatus* leaf water extract; EE: *E. alatus* leaf methanol extract; EE: *E. alatus* leaf ethanol extract. Different lowercase letters were determined using Duncan’s multiple range test (*p* < 0.05) and indicate differences between each group.

## Data Availability

The data presented in this study are available on request from the corresponding author.
